# Minutes of the Ohio Dental College Association

**Published:** 1858-03

**Authors:** J. Taft


### MINUTES OF THE OHIO DENTAL COLLEGE ASSOCIATION.

The Association met according to adjournment, in the Dental College, on Monday, Feb. 15, 1858, at 2 o'clock, P. M.

Present—Drs. Bonsall, Jas. Taylor, W. R. Webster, C. B. Chapman, H. R. Smith, Geo. Watt, J. Taft.

Dr. Bonsall, President, made some remarks and suggestions upon business matters, requiring the consideration of the Association.

Drs. Jos. Richardson, of Cincinnati, H. A. Smith, of Oxford, O., and J. S. King, of Pittsburg, Pa., having become stockholders, were unanimously elected members of the Association.

Dr. W. R. Webster was appointed on the examining committee, to fill the vacancy occasioned by the absence of Dr. H. Munro.

Adjourned to 3 o'clock, P. M., to-morrow.

SECOND DAY.

The Association met according to adjournment, at 3 P. M.

Present—Drs. Taylor, Watt, Chapman, Webster, Bonsall, J. B. Smith, Richardson, H. A. Smith, Ulrey.

The minutes of the first session were read and approved.

On motion of Dr. Taylor,

*Resolved*, That the interest on the stock for the next four years be appropriated to the payment of the debt now resting on the institution.

*Report of the Faculty of the Ohio College of Dental Surgery.*

The Faculty of the Ohio College of Dental Surgery respectfully present the following report of the session about to close:

In accordance with the nomination made at the last meeting of this Society, the Trustees elected Joseph Richardson, D. D. S., to the chair of Mechanical Dentistry, made vacant by the resignation of H. R. Smith, D. D. S. The position was accepted by Dr. R., and he has, accordingly, sustained his department in the college the present session.

By an action of the Faculty, H. A. Smith, D. D. S., was appointed Demonstrator of Operative and Mechanical Dentistry, and has also labored with us the present session.

The Infirmary was not kept open during the summer, as the member of the class selected as resident dentist found it impracticable to remain in the institution. It will, probably, be kept open during the coming summer.

The course of instruction began, as usual, on the first Monday of November, and continued till the present time.

We have to report a slight decrease in the size of the class, owing, probably, to the influence of the financial crisis; as previous to the stringent period, the prospect was fair for an increase. We refer with pleasure to the studious habits and courteous demeanor of the present class, and are happy to report that no incident has occurred to mar the harmony of our Institution.

We present five candidates for the degree of Doctor of

Dental Surgery. These have all attended two full courses in this Institution.

The following table embraces a list of the Infirmary operations of the session:

| | |
|---|---|
| No. Full Sets Teeth inserted during the session | 5 |
| " Full Upper Sets | 12 |
| " Full Lower Sets | 9 |
| " Partial Sets | 8 |
| " Teeth filled with gold | 237 |
| " " " " other material | 59 |
| " " Extracted | 453 |
| " Cases Salivary Calculus | 48 |
| " Pivot Teeth | 4 |
| " Jobs Repairing | 10 |
| " Nerves destroyed and fangs filled | 20 |

*Names of Graduates.*

S. L. Bracey, . . . . . . . . . . . . . . . . . . . . *Mississippi.*
Wm. A. Ross, . . . . . . . . . . . . . . . . . . . . . *Ohio.*
C. C. Dills, . . . . . . . . . . . . . . . . . . . . . . *Ohio.*
M. W. Wartman, . . . . . . . . . . . . . . . . . . *Canada West.*
Charles W. Robinson, . . . . . . . . . . . . . . *Ohio.*

On motion of Dr. Watt,

*Resolved*, That we now go into an election of officers for the ensuing year, which resulted as follows:

*President*, - Dr. C. BONSALL.
1*st Vice President*, " J. P. Ulrey.
2*d* " " " W. R. Webster.
*Secretary*, - " J. Taft.
*Treasurer*, - " J. Taylor.

*Dental Examiners*—Dr. E. Collins, of Connersville, Ia.; Dr. W. W. Allport, of Chicago, Ill.; and Dr. H. A. McDaniels, of Nashville, Tenn.

*Medical Examiners*—Dr. Samuel Martin, Xenia, O.; Dr. E. B. Stevens, Cincinnati, O.

Adjourned to Thursday, 9 o'clock, A. M.

THURSDAY.

The Association met at 9 A. M.

Present—Drs. Taylor, Bonsall, Webster, Chapman, Richardson, Watt, Taft.

The Treasurer's report was presented.

The Treasurer of the Ohio Dental College Association would respectfully report that the deficit which existed at the close of the last financial year, of $199 38, was borrowed on six months' time, and this, with the interest, has again been borrowed, and will be due 5th of March. The account stands as follows:

| | | |
|---|---|---|
| *The Ohio Dental College Association to the Treasurer,* | | DR. |
| | To cash to meet deficit in Treasury, Feb. 28th, 1857 | $199 38 |
| | Interest on above for six months | 17 91 |
| Sept. 4 | Interest on renewal of note | 21 55 |
| " | Account Book | 1 00 |
| " | Balance on bill to Messrs. Lord & Wright | 10 00 |
| " | Postage and Express | 1 56 |
| | | $251 40 |
| *The Association,* CR. | | |
| | By Interest on cash in Treasurer's hands | 3 00 |
| | | $248 40 |

| | | |
|---|---|---|
| For the present year the Treasurer has received rent of College from the Faculty | | $510 00 |
| Paid bill for paving | $ 62 70 | |
| Interest on Mortgages | 400 00 | |
| | $462 70 | 462 70 |
| | | $47 30 |

Leaving $47 30, which can be used to reduce the floating debt of $251,-40, due 1st of March next.

Respectfully, etc., JAS. TAYLOR, *Treasurer.*

The above was audited, and found to be correct.

Dr. Bonsall reported that the Treasurer had given the required bond for the faithful performance of his duty.

Adjourned to meet on the third Monday of Feb., 1859, at 2 o'clock, P. M. J. TAFT, *Sec'y.*

